# Commentary: Development of a Computer-Aided Design and Finite Element Analysis Combined Method for Affordable Spine Surgical Navigation With 3D-Printed Customized Template

**DOI:** 10.3389/fsurg.2021.743290

**Published:** 2021-09-23

**Authors:** Som P. Singh, Kiera G. Borthwick, Fahad M. Qureshi

**Affiliations:** ^1^Department of Biomedical Sciences, University of Missouri–Kansas City School of Medicine, Kansas City, MO, United States; ^2^Department of Neurosciences, Washington & Lee University, Lexington, VA, United States

**Keywords:** surgical navigation, 3D-printing, spine surgery, commentary, template

## Introduction

Accurate screw placement in spinal fixation is paramount for improved surgical outcomes. Current literature suggests higher rates of complication, including damage to visceral organs, are found in screw placement procedures which do not employ additional measures of surgical navigation–opting for a more “freehand” approach (1). With regard to these additional measures, the increased availability of three-dimensional (3D) printing technology has led to this technology becoming a greater avenue for exploration in improving surgical outcomes. Historically, medical application of the 3D printing was focused on anatomical models, biomaterials, and medical instruments, primarily in orthopedics (2). Overtime, the increased availability has contributed to lower costs of 3D-printing, improving its consumer demand and research development.

Given the delicate nature of spinal anatomy, additive manufacturing (1) of 3D printed technology provides, essentially, an additional layer of stereotactic navigation that can provide patient-specific planning for each pathology, creating a potential area of focus in improving surgical outcomes.

## 3D Printing as a Patient-Specific Treatment Modality

The recent publication by Eltes et al. (3) outlines a therapeutic attempt on a patient with a history significant for multiple spinal surgeries presenting with acute broken S1 left pedicle screw in addition to associated failure of solid fusion between the L5 and S1 vertebrae. In summary, this study utilized Quantitative Computed Tomography (QCT) scans and segmentation to generate a three-dimensional geometric model of the patient's sacrum. Using Mimics^®^ image analysis software, surface mesh was generated. Each element of the mesh was assigned material properties in accordance with the bone density of the sacrum at that location. This process required two steps for the bone tissue. First, Hounsfield Units (HU) for radiodensity were converted to bone mineral density (BMD) values. The density was finally converted to elastic modulus to create the finite element patient-specific model of the sacrum. A model of the polyaxial pedicle screw was also generated. After modification to a monoaxial head, the screw was virtually inserted into the model of the patient's sacrum in a convergent (S1) and divergent (ALA) position.

Nine different finite element meshes were developed for each potential surgical scenario, each with a different mesh edge length, ranging from 2.0 to 6.0 mm. The sacrum model was fixed in place at the S1 endplate and lower portion of the sacrum. It experienced 500N of load applied to the head of the screw. The virtual meshes were printed with masked stereolithography technology out of photopolymer resin, and the sacrum was printed using Fused Deposition Modeling. The drilling template was placed onto the printed model of the sacrum, and a drill bit was drilled into both positions. The accuracy of the model was assessed via two CT scans and a 3-matic software three-dimensional measurement tool.

Ultimately, the template allowed for a patient-specific, accurate placement of the screw in both convergent and divergent positions. The drill bits were not perfectly aligned with the screws in the virtual plan, but they only varied by 4.42 and 2.4 degrees for the convergent and divergent screws, respectively.

## Discussion

The methodology provided by Eltes et al. is a sound design; however, follow up data on this patient's surgical outcomes would benefit further understanding the reliability of utilizing a 3D printed template. Given the nature of recurrent spinal pathology, this patient may have external factors which could contribute to the post-operative stress applied to the new screw (i.e., osteoporosis) (1, 3, 4).

## Notable Future Directions

With the lowering costs of 3D printing, spare copies of the 3D printed template which are originally not intended for surgical use can be used by the surgeon to inspect and grow familiar with the patient's unique anatomy prior to surgery. Furthermore, if cost still permits, additional 3D models could be designed to create an educational opportunity for medical students participating in these surgeries during clinical clerkships (5).

From a standpoint of pedicle screw placement, there is a growing amount of literature outlining similar methodologies which utilize a patient-specific spine template for placement. In addition, a future direction which Eltes et al. (3) indirectly contributes to is in utilizing 3D printing in other areas in orthopedics such as oncologic tumors (4, 6). Given the patient-specific approach of the 3D-printed templates, it may be possible to stereotactically navigate surgical resection of a tumor utilizing QCT as outlined earlier, which can potentially cause a dramatic improvement in patient outcomes if resection margins can be obtained (5).

On the other hand, the authors of this commentary envision utilizing the computer-aided design could be investigated further to see if it can be used in a relatively different area of spine surgeries: congenital deformities. Current literature on 3D printed templates have long been dominated by its use in congenital heart and vascular pathologies (7, 8). Upon observation, the methodology by Eltes et al. could be used in further decreasing pre-operative preparation among cardiovascular procedures through similar computer-aided-designs to lessen the intraoperative CT imaging discussed in aforementioned study (3).

## Conclusion

Overall, implementation of patient-specific 3D-printed templates requires a mounting amount of evidence before it is considered to be the standard of care. Despite this, the sound methodology provided by Eltes et al. (3) provides a foundation level of evidence on patient-specific 3D-printed templates, as well as create new educational opportunities to medical students, and potentially spread into other diseases requiring invasive interventions (9, 10).

## Author Contributions

All authors listed have made a substantial, direct and intellectual contribution to the work, and approved it for publication.

## Funding

SS and FQ are recipients of the Sarah Morrison Research Award at the University of Missouri–Kansas City School of Medicine.

## Conflict of Interest

The authors declare that the research was conducted in the absence of any commercial or financial relationships that could be construed as a potential conflict of interest.

## Publisher's Note

All claims expressed in this article are solely those of the authors and do not necessarily represent those of their affiliated organizations, or those of the publisher, the editors and the reviewers. Any product that may be evaluated in this article, or claim that may be made by its manufacturer, is not guaranteed or endorsed by the publisher.
